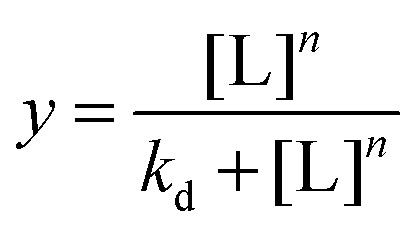# Glycosylated gold nanoparticle libraries for label-free multiplexed lectin biosensing†
†Electronic supplementary information (ESI) available. See DOI: 10.1039/c5tb01994j



**DOI:** 10.1039/c5tb01994j

**Published:** 2015-10-30

**Authors:** Sarah-Jane Richards, Lucienne Otten, Matthew I. Gibson

**Affiliations:** a Department of Chemistry , University of Warwick , Coventry , CV4 7AL , UK . Email: m.i.gibson@warwick.ac.uk ; Fax: +44 (0)2476 524112

## Abstract

Glycan/lectin interactions drive a wide range of recognition and signal transduction processes within nature. However, their measurement is complicated or limited by the analytical tools available. Most technologies require fluorescently labelled proteins (*e.g.* microarrays) or expensive infrastructure (such as surface plasmon resonance). This also limits their application in biosensing, especially for low-resource settings, where detection of pathogens based on glycan binding could speed up diagnosis. Here we employ a library-oriented approach to immobilise a range of monosaccharides onto polymer-stabilised gold nanoparticles to enable rapid and high-throughput evaluation of their binding specificities with a panel of lectins. The red to blue colour shift upon gold nanoparticle aggregation is used as the output, removing the need for labelled protein, enabling compatibility with 96-well microplates. Furthermore, we demonstrate the use of a flatbed scanner (or digital camera) to extract biophysical data, ensuring that only minimal resources are required. Finally, linear discriminant analysis is employed to demonstrate how the glyconanoparticles can be applied as a multiplexed biosensor capable of identifying pathogenic lectins without the need for any infrastructure and overcoming some of the issues of lectin promiscuity.

## Abstract 2

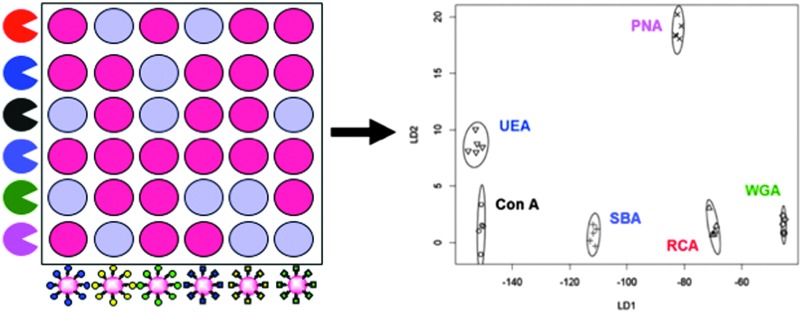
Glycosylated nanoparticle libraries are developed to enable ‘barcode’ sensing of lectins and toxins.

## Introduction

Carbohydrates coat most cell types in nature and are involved in a multitude of essential biological processes such as cell signalling, cell–cell communication, inflammation and fertilisation.^1–4^ However, bacteria, their toxins and viruses can exploit these cell surface oligosaccharides for adhesion to host tissues and is the first step in many infectious diseases.^4,5^ The proteins that mediate these interactions are known as lectins, they interact with carbohydrates non-covalently and reversibly with a high level of specificity.^6,7^ Protein–carbohydrate interactions are typically weak with values for *K*_d_ in the range of 10^–3^–10^–6^ M. This intrinsically weak protein–carbohydrate affinity is circumvented in nature by the involvement of multiple protein–carbohydrate interactions leading to the high affinity and specificity, this is known as the ‘cluster glycoside effect’, which is regarded as the enhancement in the activity of a multivalent ligand beyond the simple linear sum of the total number of ligands.^8,9^ This simple, but still not fully understood enhancement effect, presents huge opportunities in macromolecular and nanoscale science to probe glycan function.^4–6,10^

The ubiquitous nature of carbohydrate–lectin interactions in infectious diseases presents new opportunities in drug design and biosensing. For example, urinary tract infections in humans are strongly associated with *Escherichia coli* producing type I mannose specific adhesins.^5,11^ Pulmonary pathogens such as those typically infecting cystic fibrosis patients such as *Pseudomonas aeruginosa* bind to GalNAcβ1-4Gal residues on lung epithelia.^5,10^ The *Vibrio cholerae* bacteria responsible for cholera releases an AB_5_ toxin (cholera toxin) that binds to galactose containing GM1 ganglioside on intestinal epithelial cells.^12–14^ Ricin is a toxic lectin found in castor beans from the plant *Ricinus communis*, which is lethal and poses a potential security threat as a biological warfare agent.^15,16^ Also, the nature of cell-surface carbohydrates can differ considerably between diseased and normal cells.^17^ Differences in glycosylation have been detected in many cancer cells and it has been suggested that these differences determine the metastatic potential of tumours. Unique glycan markers of diseased cells can be exploited for early diagnosis, prevention and treatment of illnesses.^17^ Therefore, rapid detection of various lectin types is required, not only for diagnosis but also for developing effective therapeutics. However, glycans are inherently complex and it is observed that lectins are capable of binding a range of related carbohydrate structures to varying extents, further complicating the challenge of assigning protein–carbohydrate interactions.^3^ Carbohydrate microarrays have been developed to aid in the identification of protein–carbohydrate interactions as new drug targets,^18–21^ however, they are difficult to construct and require a protein-labelling step, which results in heterogeneity, synthetic complexity and is not a truly native assay. Therefore the development of fast, label-free, easy and inexpensive sensors for the analysis of carbohydrate–protein interactions is highly desirable. The current best methods for this are interferometry, quartz crystal microbalance (QCM) and surface plasmon resonance (SPR). However, all three of these require infrastructure and are still relatively low-throughput, running 10's of samples per hour typically.^22^

Metal nanoparticle-based materials are appealing alternatives to fluorescence- or radiolabelled protein substrates, owing to their photo-stability, ease of synthesis and ability to conjugate to biological molecules.^23,24^ Gold nanoparticles (AuNPs) are highly attractive as biosensors, due to exhibiting an intense colour in the visible region as a result of collective oscillation of the conduction-band electrons of the gold core.^23,24^ Factors affecting the optical properties of nanoparticles include, size and shape, degree of aggregation and the dispersion medium.^16^ Aggregation of AuNPs leads to a dramatic colour change from red to blue (dependant upon interparticle distance) due to electric dipole–dipole interactions and coupling between the plasmons of neighbouring particles resulting in a broadening and a shift to longer wavelengths of the surface plasmon absorption band. The colour changes associated with AuNP aggregation has been exploited in the development of colourimetric assays for biomolecular interactions by utilising AuNPs functionalised with biomolecules such as proteins, peptides and DNA.^25–29^

Carbohydrate-functionalised AuNPs (glycoAuNPs) are emerging as important tools for the colourimetric determination of carbohydrate–protein interactions as they constitute a good biomimetic model of carbohydrates at the cell surface (glycocalyx).^23,30–33^ The multivalent presentation of carbohydrates can compensate for the low affinity of individual protein–carbohydrate interactions and the inherent multivalency of lectins should lead to an optical response as a result of aggregation if the correct lectin–carbohydrate paring is present. GlycoAuNPs have been exploited for the detection of lectins,^16,32,34,35^ bacteria^31,36^ and influenza.^37^ Whilst appealing scaffolds, glycosylated nanoparticles have intrinsic low colloidal stability, resulting in either false-positive aggregation responses or preventing high-throughput application. Gibson *et al.* have recently demonstrated that through precision macromolecular engineering using RAFT (reversible addition–fragmentation chain transfer) polymerisation it is possible to improve colloidal stability, without presenting a steric block to aggregation.^35^

Herein, we describe a polymer-stabilised, glycosylated gold nanoparticle platform for the high-throughput and importantly label-free screening of carbohydrate–lectin interactions and demonstrate its potential in glycobiology in a range of assays. A mix-and-match synthetic strategy enables huge chemical space to be explored, with the outputs being read in a simple multi-well plate format by both a microplate reader, or simply using a digital camera. Through the use of a training algorithm, with linear discriminant analysis (LDA) it was possible to develop a platform that can correctly identify six distinct lectins from an unknown sample based solely on colourimetric readouts without the need for a protein labelling step.

## Results and discussion

**Scheme 1:**
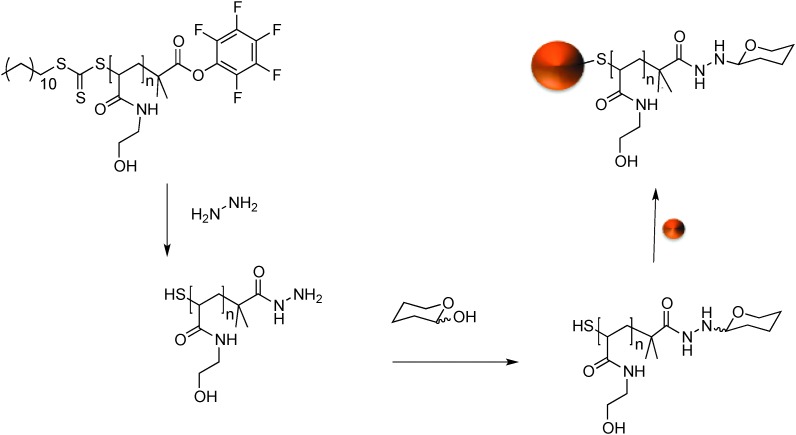
Synthetic route to carbohydrate functionalised gold nanoparticles.

GlycoAuNPs were prepared using an optimised polymer coating method we developed recently that produces particles that are stable at physiological salt concentration and gives fast lectin aggregation.^35^ Our synthetic methodology was designed to enable facile and versatile conjugation of the carbohydrates to the gold nanoparticles using native, underivatised carbohydrates, with the aim of circumventing the need for ‘click’ type conjugation and the synthetic burden of the introduction of alkynes, azides or thiols.^38,39^ Scheme 1 shows the general method employed. *N*-Hydroxyethyl acrylamide (HEA) was polymerised by RAFT polymerisation to a targeted degree of polymerisation (DP) of 20, which we have previously shown to give the precise balance between colloidal stability and rapid sensing.^35^ By using a pentafluorophenyl (PFP) containing RAFT agent, the resulting telechelic polymers had both an amine-reactive ω-terminus (for conjugation to sugars) and a protected thiol at the α-terminus for subsequent gold particle immobilisation, Scheme 1. The PFP functionality is extremely reactive to amines and has good aqueous stability, compared to NHS esters.^40,41^ The PFP moiety can also be used to install a hydrazide end group for subsequent amidation with reducing sugars.^42^ This approach enables the introduction of a range of carbohydrates, which facilitates our later analysis (*vide infra*). During carbohydrate coupling, the trithiocarbonate end-group is aminolysed releasing the thiol enabling coating of 60 nm AuNPs by simple mixing. 60 nm particles were chosen, as we have previously found that smaller particles give lower colourimetric responses.^36^ This post-polymerisation route ensures that all the polymers have the same initial chain length distribution and therefore reduced the variability between particles, but allows versatile end group functionalisation to make libraries of a variety of glycan functional particles.

The intrinsic reactivity of reducing sugars toward hydrazides to form stable *N*-glycoside end-terminated polymers was used. The reducing sugars react with α-heteroatom nucleophiles (*e.g.* hydrazide or alkylaminooxy groups) to give mostly cyclic pyranosides, but some ring-opened forms will still form. It should be highlighted that the analysis we use later (*vide infra*) is not dependant on this fact.^42^ AuNPs were functionalised with a library of seven monosaccharide-terminated polymers; mannose (Man), galactose (Gal), glucose (Glc), *N*-acetylmannosamine (ManNAc), *N*-acetylgalactosamine (GalNAc), *N*-acetylglucosamine (GlcNAc) and fucose (Fuc). Due to the high-throughput nature of this study and the small sample size generated XPS (X-ray photoelectron spectroscopy) was used to confirm the presence and nature of the polymer coating. The inclusion of the hydrazide moiety on to the polymer end could be seen by the hydrazide peak in the XPS and determined to be in the ratio of [1] : [19] end-group to repeat units, which is in close agreement with the number average degree of polymerisation which is 20. Complete spectra are included in the ESI.† Using DLS (dynamic light scattering) a 5 nm increase in particle size was noted following addition of each polymer onto the AuNPs further confirming successful functionalisation. Based on our previous studies of ‘grafting to’ gold nanoparticles from RAFT, we have seen grafting densities of ∼0.3 chains nm^–2^. On a (spherical) 60 nm particle, this would correspond to ∼3000 chains per particle, which is more than sufficient to observed the cluster glycoside effect.^43^ It should be noted that this is an estimate and that the volumes used in this study, in a combinatorial fashion, were not in sufficient volume for *e.g.* thermogravimetric analysis.

**Fig. 1:**
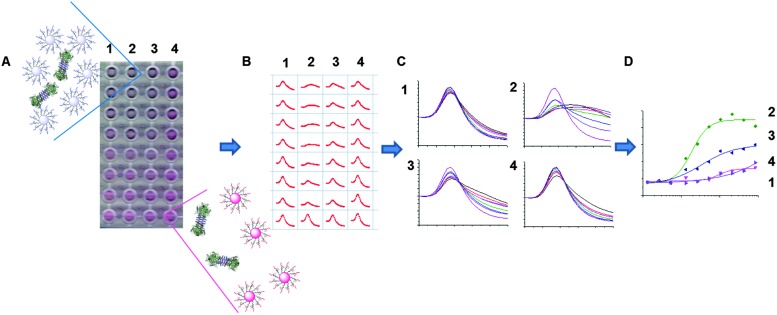
Overview of the approach used to allow direct measurement of carbohydrate–lectin interactions by monitoring a colourimetric change (red to blue) due to aggregation of glycoAuNPs bound to lectins (A) and the use of absorbance spectra (B and C) in the formation of binding curves (D).

With this library at hand, the evaluation of the AuNP interactions with a panel of six lectins was conducted; concanavalin A (Con A), *Ricinus communis* agglutinin (RCA_120_, which is a non-toxic variant of ricin), soybean agglutinin (SBA), peanut agglutinin (PNA), wheat germ agglutinin (WGA) and *Ulex europaeus* agglutinin (UEA). To assess the binding interaction, the glycosylated AuNPs were added to 96-well plate at a concentration of 0.06 M of Au. The appropriate lectins were then added as a dilution series into these wells, and incubated for 30 minutes. After this time, if binding was occurring the particles will crosslink (aggregate) causing a red to blue shift, as shown in the digital photograph of an example plate shown in Fig. 1A, where high concentrations of lectins cause an increasing red shift. This can be monitored using a UV-Vis microplate reader, to give a full spectrum for each sample, Fig. 1B, which can then be collated to visualise the overall change in as shown in Fig. 1C. For example, the spectra in Fig. 1C(1) show a sample that has not interacted with the lectin, but in Fig. 1C(2) there is a clear dose dependant response. These spectra can then be converted into binding isotherms, based on the change in absorbance at 700 nm, Fig. 1D.

**Fig. 2:**
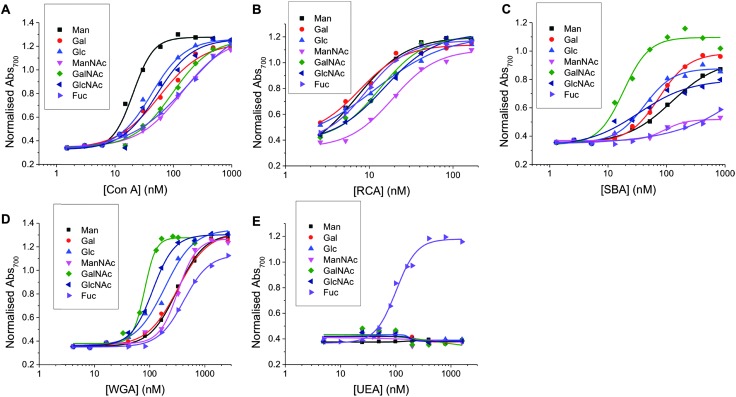
Dose-dependent binding isotherms of Man, Gal, Glc, ManNAc, GalNAc, GlcNAc and Fuc functionalised particles with (A) Con A (B) RCA_120_ (C) SBA (D) WGA (E) UEA.

Using the methodology outlined above, the serial dilutions of each lectin with each nanoparticle was conducted and analysed. In this set, a total of 280 individual, label-free, interaction measurements were conducted (7 sugars, 5 lectins, 8 concentrations) (ESI†). Fig. 2 summarises the data as dose-dependant binding isotherms for all of these, by plotting the Abs_700_ against protein concentration. As can clearly be seen, a vast amount of data is readily obtained by this method, which would be challenging, expensive and time-consuming to extract from SPR type methods, for example. It should be noted that PNA (peanut agglutinin) was also used, but the data was inconsistent and appeared to be a protein stability issue. Therefore, this data is not included. Incubation of non-glycan functionalised nanoparticles (made from a carboxyl-functional RAFT agent) showed no change in colour, as would be expected for a protein resistant coating such as pHEA.

To enable these curves to be used to understand their binding interactions, all the data were fitted to Hill functions to obtain the apparent dissociation constants (*K*_d apparent_) as a measure of the relative affinity of each glycan to the lectin partner. These values are summarised in Table 1. The selectivity of each lectin for monosaccharide agreed well with microarray data from the Consortium for Functional Glycomics (CFG) database.^44^ It is important to highlight that this data shows that monosaccharides (and even more complex glycans) do not bind a single lectin (and that any given lectin can bind many different carbohydrates) and that it is crucial to not study these interactions in isolation, but as a panel of lectins in order to truly probe specificity and affinity. For example, SBA is β-*N*-acetylgalactosamine specific and shows the highest affinity to GalNAc-functional particles and Con A is α-mannose/α-glucose specific and shows the highest affinity for Man-functional particles and UEA is fucose specific and interacts highly specifically with Fuc-functional particles and showed essentially zero binding to other carbohydrates in the concentration range used here. No interaction is observable for any lectins across the concentration range used here for polymers without a carbohydrate end-group. This broad set of data also shows the challenges intrinsic to developing biosensors based on glycans, due to their broad binding potential that makes specific ‘lock and key’ style interactions challenging. This particular point is addressed later in this manuscript.

**Table 1:** Dissociation constant (*K*_d apparent_) in nM for each glycoAuNP and lectin combination determined by Abs_700_. N/A means they showed no interaction in the concentration range used

	Con A	RCA_120_	SBA	WGA	UEA
Man	20.4 ± 1.6	7.6 ± 2.5	124.5 ± 16.5	317.3 ± 16.1	N/A
Gal	58.7 ± 8.7	7.1 ± 3.3	74.6 ± 4.2	319.9 ± 26.4	N/A
Glc	42.2 ± 3.8	11.7 ± 3.6	42.0 ± 3.7	187.7 ± 20.5	N/A
ManNAc	140.2 ± 22.3	19.0 ± 1.9	92.8 ± 22.7	326.2 ± 24.0	N/A
GalNAc	96.6 ± 11.1	13.4 ± 2.3	18.5 ± 2.4	80.1 ± 4.4	N/A
GlcNAc	52.2 ± 9.0	15.1 ± 1.5	33.7 ± 11.8	113.5 ± 4.4	N/A
Fuc	177.8 ± 33.9	9.2 ± 0.8	N/A	415.6 ± 24.2	100.5 ± 6.9

### Measuring interactions using a scanned image

**Fig. 3:**
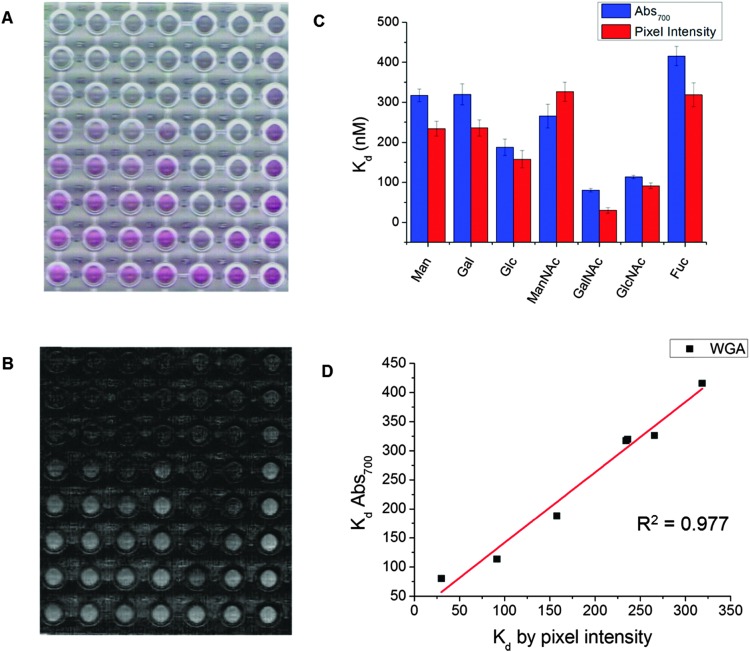
Direct optical analysis of lectin/glycan interaction. (A) Scanned image of glycoAuNPs with a dilution series of WGA after 30 minutes incubation at 37 °C. (B) Saturation image used for pixel intensity measurement (high intensity = red, low intensity = blue). (C) Comparison of *K*_d_ calculated by Abs_700_ and pixel intensity. (D) Correlation between *K*_d_ values determined by Abs_700_ and pixel intensity.

Due to the strong colouration of the gold particle solutions, the colour change upon binding is obvious to the naked eye (Fig. 1A). Previously, we demonstrated that citrate stabilised-AuNPs could be used as resolving agents in sorbent assays of lectins to carbohydrate functional 96-well plates by measuring the amount of non-specifically bound AuNPs to a lectin bound on the surface, and that a simple mobile phone camera, and image analysis software could be used to measure inhibition.^45^ Due to the high extinction coefficient of AuNPs we employed a similar technique here to determine the degree of aggregation and hence affinity from a scanned image of the 96-well plate (Fig. 3A) and simple, open access visualisation software, ImageJ to obtain pixel intensity (Fig. 3B). Fig. 3C shows the comparison of *K*_d_ value determined by UV-Vis and by pixel intensity. Fig. 3D shows the correlation between the *K*_d_ values determined by the two methods. It shows strong positive correlation between the techniques with an *R* ^2^ value of 0.977, demonstrating that this analysis is also possible in laboratories without a microplate reader, or in low resource settings. All extracted *K*_d apparent_ values are also given in Table 2.

**Table 2:** Dissociation constant (*K*_d apparent_) in nM for each glycoAuNP and lectin combination determined by pixel intensity from a scanned image. N/A means they showed no interaction in the concentration range used

	Con A	RCA_120_	SBA	WGA	UEA
Man	18.2 ± 1.2	4.7 ± 0.4	97.6 ± 9.1	234.2 ± 18.3	N/A
Gal	54.4 ± 9.0	1.5 ± 0.1	62.2 ± 22.3	235.9 ± 20.3	N/A
Glc	36.7 ± 6.9	5.5 ± 1.6	57.4 ± 11.6	157.8 ± 22.0	N/A
ManNAc	109.4 ± 10.6	16.1 ± 1.5	N/A	265.6 ± 29.6	N/A
GalNAc	70.1 ± 6.0	7.5 ± 0.4	11.2 ± 0.8	29.8 ± 7.2	N/A
GlcNAc	49.1 ± 14.1	7.9 ± 0.4	30.5 ± 4.4	91.5 ± 6.8	N/A
Fuc	169.5 ± 32.9	6.6 ± 0.6	N/A	318.6 ± 39.7	42.6 ± 3.3

### Differentiation of lectins

**Fig. 4:**
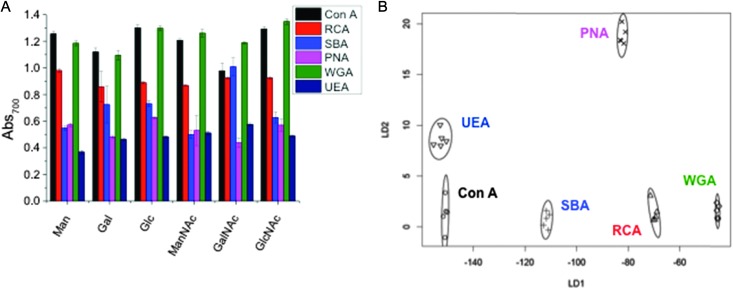
Lectin discrimination. (A) Absorbance at 700 nm of Man, Gal, Glc, ManNAc, GalNAc and GlcNAc functionalised particles with 6.25 μg mL^–1^ Con A, RCA_120_, SBA, WGA, PNA, UEA. Each is the average of 5 measurements. (B) Linear discriminant plot showing excellent segregation between lectins.

As a final demonstration of the utility of this methodology, we sought to apply the rapid colour change as a versatile lectin discrimination tool, which could be used in diagnostics. Due to the relatively low specificities of monosaccharides to lectins (compared to complex glycans), glycoconjugates often show a broad range of binding affinities to several lectins (as exemplified in Table 1, above). However, we have previously shown that by replacing a single glycoconjugate, with a panel of them, it is possible to use multiplexed (or ratiometric) assays to discriminate between a panel of fluorescently labelled Gal-specific lectins as a ‘chemical tongue’, based on the differences in relative affinity between sugars.^46^ This work also showed that in a ‘real application’ the sample would not be a purified protein this approach could also detect lectin mixtures, when the lectin of interest was present in more than 50% by mass. We therefore reasoned that the colour-changing assay described here could be extended to a label free diagnostic platform. Hence, a range of six glyconanoparticles, were incubated with our panel of six lectins, and the total change in Abs_700_ recorded, and plotted in Fig. 4A. As can be clearly seen, any individual lectin binds multiple sugars, and *vice versa*. However, the pattern of binding is unique to each lectin, like a barcode or fingerprint. Linear discriminant analysis (LDA) was applied to generate the plot shown in Fig. 4B. LDA takes an input training matrix of the initial particles bound to the different glyconanoparticles (the data displayed in Fig. 4A) and generates distinct binding profiles for all the lectins with maximum separation between groups in order to generate a robust model for identifying unknown lectin samples without any fluorescent labels (Fig. 4B).

Fig. 4B clearly shows that excellent segregation between each lectin is achieved. Using a leave-one-out approach, where a model is produced from the rest of the data, all lectins can be correctly identified with a high level of confidence. The minimum variables required to correctly predict the lectins can be determined. In this instance, there are a number of two glycoAuNP pairings that will correctly predict all the lectins, including Man and GalNAc or GlcNAc and GalNAc, however, the certainty decreases with fewer variables. This glycoAuNP technique therefore constitutes a robust and versatile platform for investigating carbohydrate–protein interactions and can also be used to identify lectin samples.

## Conclusions

Here a new and easy to use platform for assessing carbohydrate–lectin interactions is demonstrated based on the aggregation of polymer-stabilised, glycosylated gold nanoparticles in response to unlabelled proteins. The construction of the particles is crucial, with the precise macromolecular features of the polymer coating being essential to provide assay readout, but simultaneously ensuring colloidal stability. The versatility of this was demonstrated by the immobilisation of seven sugars, plus measurement of their binding to six distinct lectins at eight concentrations enabling >300 individual measurements. This was achieved with only the use of low volume 96 well plates and UV-Visible plate reader by monitoring the colourimetric changes associated with gold nanoparticle aggregation. Binding isotherms were extracted to allow estimation of binding affinities, and it is hoped that such a method will provide an easy to access alternative to conventional surface plasmon resonance methods that require expensive infrastructure. To further simplify the procedure, it is demonstrated that the colourimetric changes can also be interpreted by using only a simple flatbed scanner and open access image analysis software, to remove the need even for a microplate reader, making this a truly accessible method.

Due to the high density of data that is accessible, in a short period of time, a multiplexed lectin discriminatory assay (‘chemical tongue’) was established. By monitoring the relative changes in aggregation state of a range of glyconanoparticles to one of six lectins, a training algorithm was developed, based on linear discriminant analysis. This analysis enables identification between lectins with similar carbohydrate-binding preferences, based on their multiplexed response. Such an easy assay, removes the need for complex, expensive and inaccessible branched glycans, or antibodies, and only uses monosaccharides to extract protein characterisation data and may find use in low resource settings for the identification of pathogens such as cholera.

## Materials

All chemicals were used as supplied unless otherwise stated. Acetone, dichloromethane, toluene, methanol, diethyl ether and dimethylformamide were purchased from Fischer Scientific at laboratory grade. Dodecane thiol (≥98%), potassium phosphate tribasic (≥98%), carbon disulfide (99%), *N*-hydroxethyl acrylamide (97%), 4,4′-azobis(4-cyanovaleric acid) (98%), mesitylene (reagent grade), *N*-(3-dimethylaminopropyl)-*N*′-ethylcarbodiimide hydrochloride (≥98%), hydrazine hydrate solution (78–82%) d-(+)mannose, α-d-glucose, d-(+)galactose, d-galactosamine hydrochloride (98%), d-mannosamine hydrochloride (98%), d-glucosamine hydrochloride (<98%) and *N*-acetyl-d-mannosamine were all purchased from Sigma-Aldrich. 2-Bromo-2-methylpropionic acid (98%), 4-(dimethylamino)pyridine (99%), pentafluorophenol (99%), triethylamine (99%) were purchased from Acros. Low bind, low volume, clear, flat bottom, microtitre plates were purchased from Greiner Bio-one. *N*-Acetylgalactosamine, *N*-acetylglucosamine and fucose were purchased from Carbosynth. 10 mmol HEPES buffer containing 0.15 M NaCl, 0.1 mM CaCl_2_ and 0.01 mM MnCl_2_ (pH 7.5, HEPES) was prepared in 200 mL of MilliQ water (with a resistance >19 mOhms). 60 nm gold nanoparticles were obtained from BBI Solutions. Concanavalin A, soybean agglutinin, *Ricinus communis* agglutinin, peanut agglutinin, wheat germ agglutinin and *Ulex europeaus* agglutinin were purchased from Vector Labs.

## Methods

### End group modification of PFP-polyhydroxyethyl acrylamide using hydrazine

PFP-pHEA (500 mg, 0.18 mmol), hydrazine (50 μL, 1.5 mmol) were dissolved in 5 mL DMF. The reaction was stirred at 50 °C for 16 h. The polymer was precipitated into diethyl ether from methanol three times and dried under vacuum. IR indicated loss of C=O stretch corresponding to the PFP ester.

### Carbohydrate functionalisation of hydrazide-pHEA

In a typical reaction, hydrazide-pHEA (10 mg, 0.0043 mmol), reducing sugar (3 mg) in 1 mL 100 mM acetate containing 1 mM aniline. Left at 50 °C over night. Employed immediately for gold nanoparticle functionalisation.

### Gold nanoparticle functionalisation using a carbohydrate terminated pHEA

1 mg of polymer in 100 μL water was added to 1 mL of 60 nm particles (OD_600_ = 1, 0.288 mM Au) and left for 30 minutes at room temperature. Following incubation the particles were centrifuged at 6000 rpm, the supernatant was removed along with any unattached polymer, the particle were then resuspended in water.

### Lectin induced aggregation studies by absorbance

A 0.1 mg mL^–1^ stock solution of the lectin was made in 10 nM HEPES buffer with 0.05 M NaCl, 0.1 mM CaCl_2_ and 0.01 mM MnCl_2_. 25 μL serial dilution was made up in the same buffer in a 96-well micro-titre plate. 25 μL of the glycoAuNP were added to each well. After 30 minutes an absorbance spectrum was recorded from 450–700 nm with 10 nm intervals. The value at 700 nm was normalised by the value at 450 nm and binding isotherms were constructed in OriginPro 9.1 using the hill function which is described by eqn (1). Where L is the lectin concentration, *K*_d_ is the apparent dissociation constant and *n* is the Hill coefficient which describes the binding cooperativity.
1