# Supplementary material for: Glycosylated gold nanoparticle libraries for label-free multiplexed lectin biosensing
Source: J Mater Chem B. 2015 Oct 30;4(18):3046–53. doi: 10.1039/c5tb01994j (PMC4859411; doi:10.1039/c5tb01994j)
Supplement: Supplementary file 1 [file TB-004-C5TB01994J-s001.pdf]

Electronic Supplementary Material (ESI) for Journal of Materials Chemistry B.
This journal is © The Royal Society of Chemistry 2015

Supporting Information For;

Glycosylated Gold Nanoparticle Libraries for Label-Free Multiplexed Lectin Biosensing

Sarah-Jane Richards,[a] Luciene Otten and Matthew I. Gibson [a,*]

[a] Department of Chemistry, University of Warwick, Coventry, CV4 7AL

*Corresponding Author Information

Fax: +44(0)2476 524112;

E-mail : m.i.gibson@warwick.ac.uk

Methods

Synthesis of 2-(Dodecylthiocarbonothioylthio)-2-methylpropionic acid (DMP) –

Dodecane thiol (4.00 g, 19.76 mmol) was added dropwise to a stirred suspension of K_3PO_4 (4.20g, 19.76 mmol) in acetone (60 mL) over 25 minutes. CS_2 (4.10 g, 53.85 mmol) was added and the solution turned bright yellow. After stirring for ten minutes 2-bromo-2-methylpropionic acid (3.00 g, 17.96 mmol) was added and a precipitation of KBr was noted. After stirring for 16 hours, the solvent was removed under *in vacuuo* and the residue was extracted into DCM (2 x 200 mL) from 1M HCl (200 mL). The organic extracts were washed with water (200 mL) and brine (200 mL) and further dried over $MgSO_4$. The solvent was removed under reduced pressure and the residue was purified by column chromatography on silica using an eluent comprising 75:24:1 40 – 60 °C petroleum ether: diethyl ether: acetic acid to yield a bright yellow solid (4.01 g, 61 %).

^1H NMR (300 MHz, $CDCl_3$) δ_{ppm}: 3.26 (2H, t, $J_{12\text{-}11}$ = 7.40 Hz, H^{12}); 1.70 (6H, s, H^{13}); 1.65 (2H, m, H^{11}); 1.36 (2H, m, H^{10}); 1.23 (16H, br. s, $H^{2\text{-}9}$); 0.86 (3H, t, $J_{1\text{-}2}$ = 6.5 Hz, H^1).

^{13}C NMR (500 MHz, $CDCl_3$) δ_{ppm}: 220.19 (C^{13}); 177.76 (C^{16}); 54.91 (C^{14}); 36.46 (C^{12}); 31.31, 29.02, 28.95, 28.84, 28.74, 28.50, 28.36, 22.08 ($C^{2\text{-}9}$); 28.59 (C^{10}); 27.19 (C^{11}); 24.60 (C15); 13.52 (C^1).

IR ν: 2921 (CH_2); 1714 (C=O); 1070 (S-(C=S)-S) cm^{-1}.

Synthesis of Pentafluorophenyl 2-(Dodecylthiocarbonothioylthio)-2-methylpropionic acid (PFP-DMP) –

DMP (0.5 g, 1.37 mmol), N-(3-Dimethylaminopropyl)-N′-ethylcarbodiimide hydrochloride (EDC) (0.39 g, 2.05 mmol), and 4-(dimethylamino)pyridine (DMAP) (0.25 g, 2.05 mmol) in

40 mL DCM was stirred for 20 minutes under N_2. Pentafluorophenol (0.78 g, 4.24 mmol) in 5 mL DCM was added. The reaction was stirred overnight at room temperature. The reaction was washed successively with 3 M HCl, 1 M $NaHCO_3$ and 0.5 M NaCl. The reaction was then dried over $MgSO_4$, filtered and then concentrated *in vacuuo*.

^{1}H NMR (300 MHz, $CDCl_3$) δ_{ppm}: 3.29 (2H, t, $J_{12\text{-}11}$ = 7.3 Hz, H^{12}); 1.84 (6H, s, H^{13}); 1.67 (2H, q, J = 7 Hz, H^{11}); 1.37 (2H, m, H^{10}); 1.23 (16H, br. s, $H^{2\text{-}9}$); 0.86 (3H, t, $J_{1\text{-}2}$ = 6.5 Hz, H^1).

^{13}C NMR (500 MHz, $CDCl_3$) δ_{ppm}: 220.19 (C^{13}); 169.77 (C^{16}); 54.91 (C^{14}); 36.46 (C^{12}); 31.31, 29.02, 28.95, 28.84, 28.74, 28.50, 28.36, 22.08 ($C^{2\text{-}9}$); 28.59 (C^{10}); 27.19 (C^{11}); 24.60 (C15); 13.52 (C^1).

IR ν: 2921 (CH_2); 1779 (C_6F_5C=O); 1070 (S-(C=S)-S) cm^{-1}.

Polymerisation of hydroxyethyl acrylamide using PFP-DMP DP20

N-Hydroxyethyl acrylamide (HEA) (0.5 g, 4.34 mmol), Pentafluorophenyl 2-(Dodecylthiocarbonothioylthio)-2-methylpropionic acid (PFP-DMP) (0.115 g, 0.22 mmol), 4,4′-Azobis(4-cyanovaleric acid) (ACVA) (0.0122 g, 0.043 mmol were dissolved in 50:50 Toluene:Methanol (4 mL). Mesitylene (150 μL) was added as an internal reference. An aliquot was taken for NMR analysis in $CDCl_3$. The solution was degassed under N_2 for 30 mins. The reaction was stirred at 70 °C for 90 mins. An aliquot was taken for NMR analysis in MeOD. The reaction was rapidly cooled in liquid nitrogen and precipitated into diethyl ether. The polymer was reprecipitated into diethyl ether from methanol twice to yield a yellow polymer product that was dried under vacuum. 90 % conversion by NMR, M_n (Theoretical) = 2800 g.mol^{-1} M_n (SEC) = 4100 g.mol^{-1} M_n/M_w (SEC) = 1.14.

Particle size determination by Dynamic Light Scattering (DLS)

400 µL of glycoAuNPs in a disposable low volume cuvette. 3 measurements comprised of 10 runs were made of each sample at 25 °C. Average size and distribution were recorded.

Lectin induced aggregation studies by pixel intensity

The plates were then scanned using a canon flatbed scanner, the images were converted to a HSB stack and the saturation image was used for tiff image file uploaded into the open-source image processing package ImageJ (version 1.46a) where a region of interest was drawn around every well. The colour (RGB) image was then converted into a hue saturation and brightness (HSB) stack of images and the saturation image used. The regions of interest drawn on the original image were added to the saturation image using the ROI manager and average pixel intensity in each region of interest was measured using an inbuilt function in ImageJ.

Linear discriminant analysis

A fixed concentration of 6.25 $\mu g.mL^{-1}$ lectin was added to glycoAuNPs. 5 repeats were made for each carbohydrate functionalised particle and the absorbance at 450 nm and 750 nm were recorded for linear discriminant analysis.

Every lectin was added to every surface as described in the lectin binding assay section. This was repeated 4 times to generate a training data matrix of 6 particles x 6 lectins x 5 replicates, which was then subjected to a classical linear discriminant analysis (LDA) in R (version 2.14.1).

Additional Figures

Size determination of goldnanoparticles by DLS. Before functionalisation (60 nm) and after functionalisation with carbohydrate terminated polymers. There is a size increase of about 5 nm.

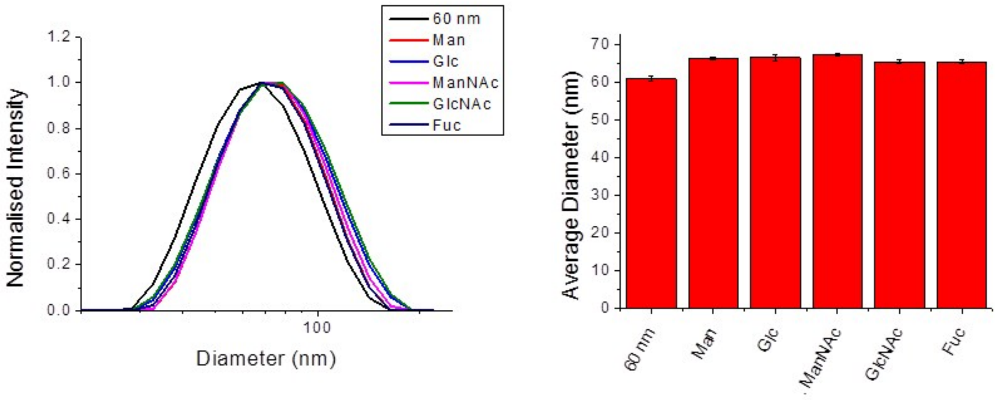

Figure S1: DLS characterisation of particles before and after functionalisation with carbohydrate functionalised polymers

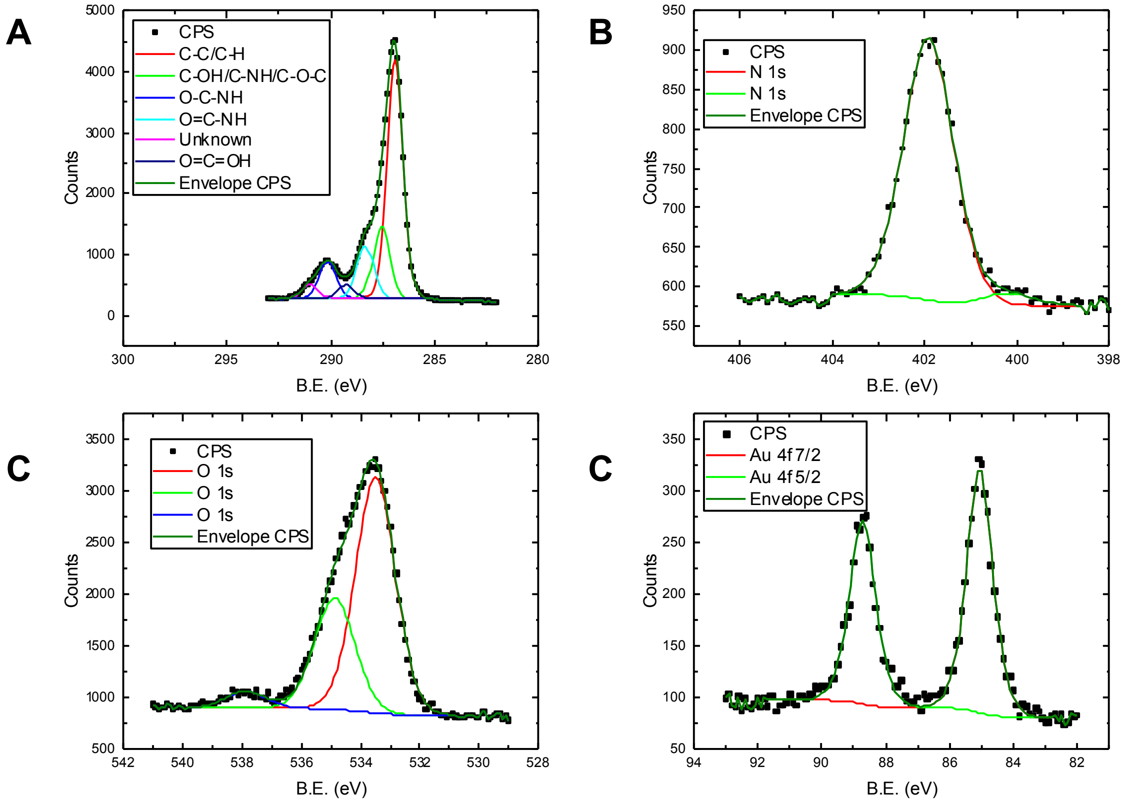

Figure S2: XPS A) C1s B) N1s C) O1s D) Au4f

K_d values for each lectin on each surface derived from the hill plots.

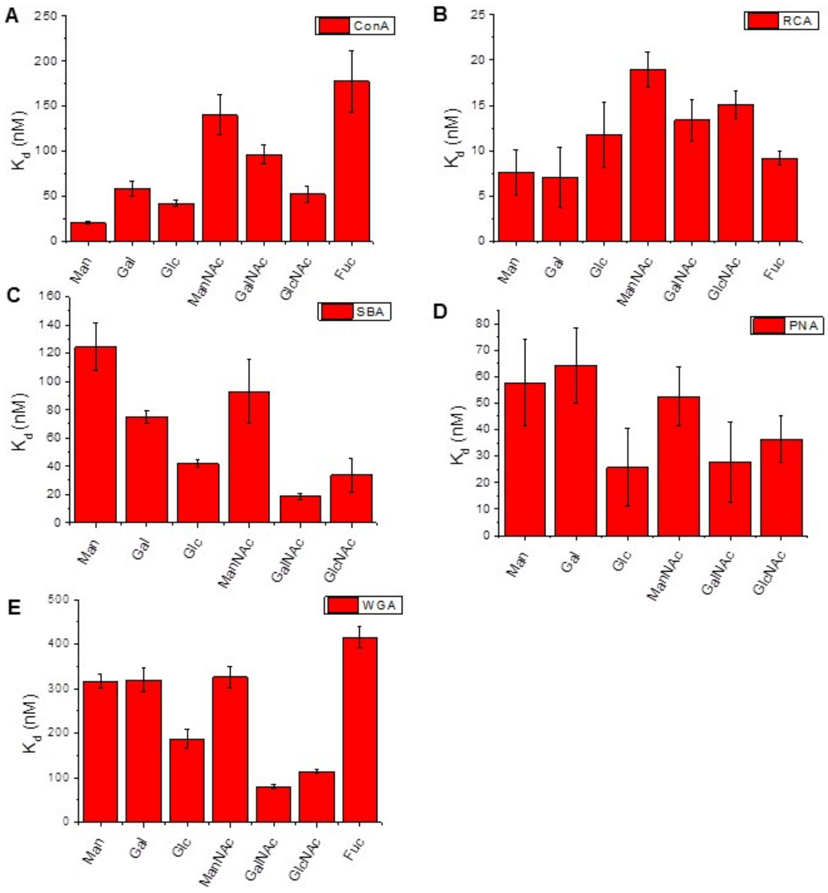

Figure S3: $K_{d\ apparent}$ values for all carbohydrate functionalised particles with A) Con A, B) RCA_{120}, C) SBA, D) PNA and E) WGA. This the graphical representation of Table 1 from the main article.

Size increase of over time of glycogoldnanoparticles upon addition of 200 nM SBA.

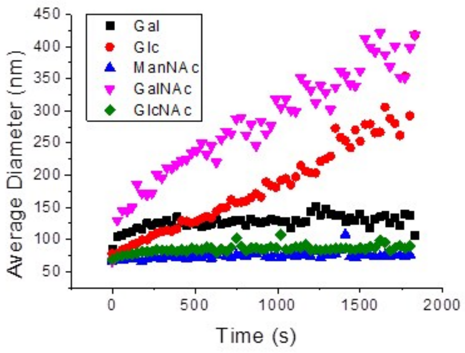

Figure S4: DLS: Kinetic increase in particle size due to aggregation induced by addition of 200 nM SBA.

Absorbance spectra for each glycoAuNP and lectin paring.

Mannose functionalised particles

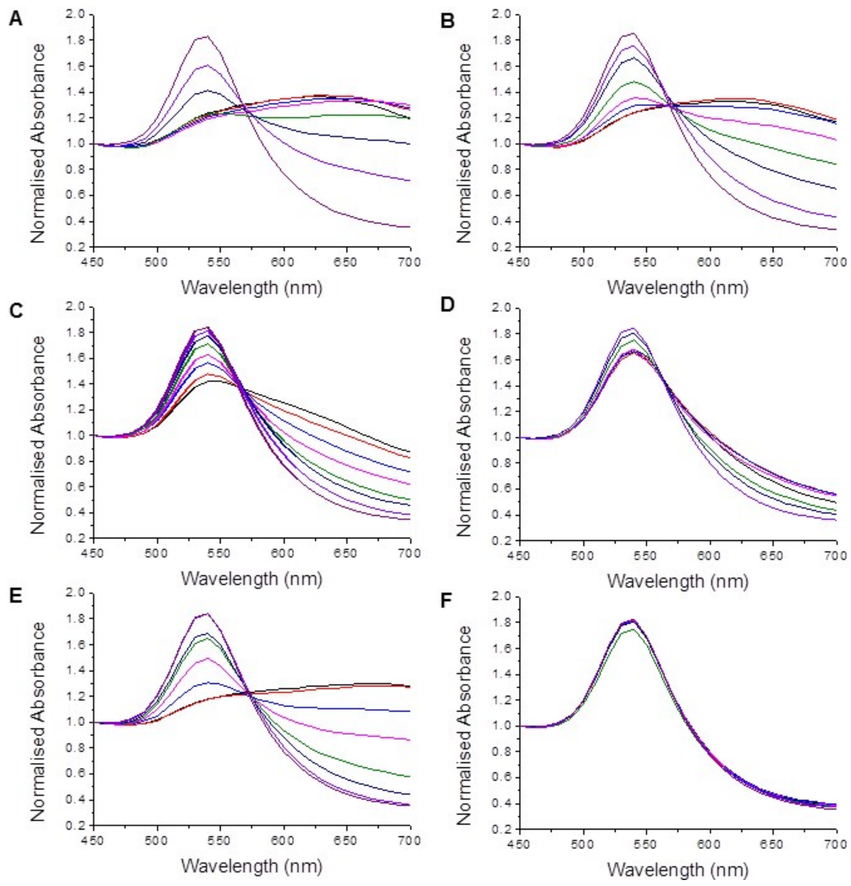

Figure S5: Man-AuNPs with A) Con A, B) RCA, C) SBA, D) PNA, E) WGA, F) UEA.

Galactose functional particles

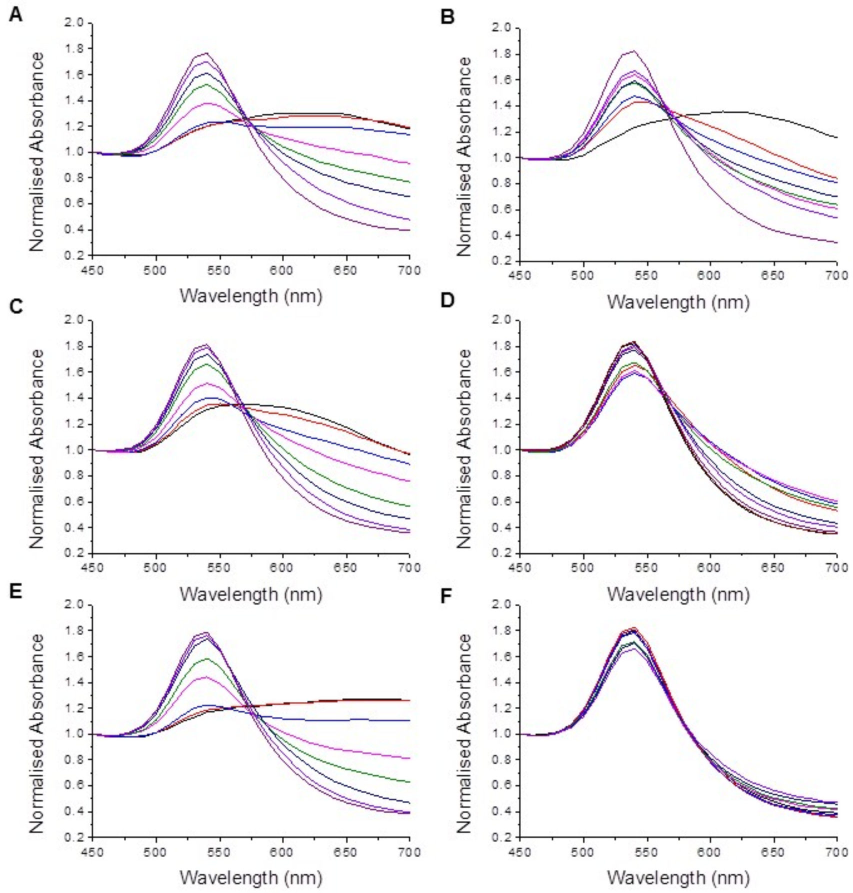

Figure S6: Gal-AuNPs with A) Con A, B) RCA, C) SBA, D) PNA, E) WGA, F) UEA.

Glucose functional particles

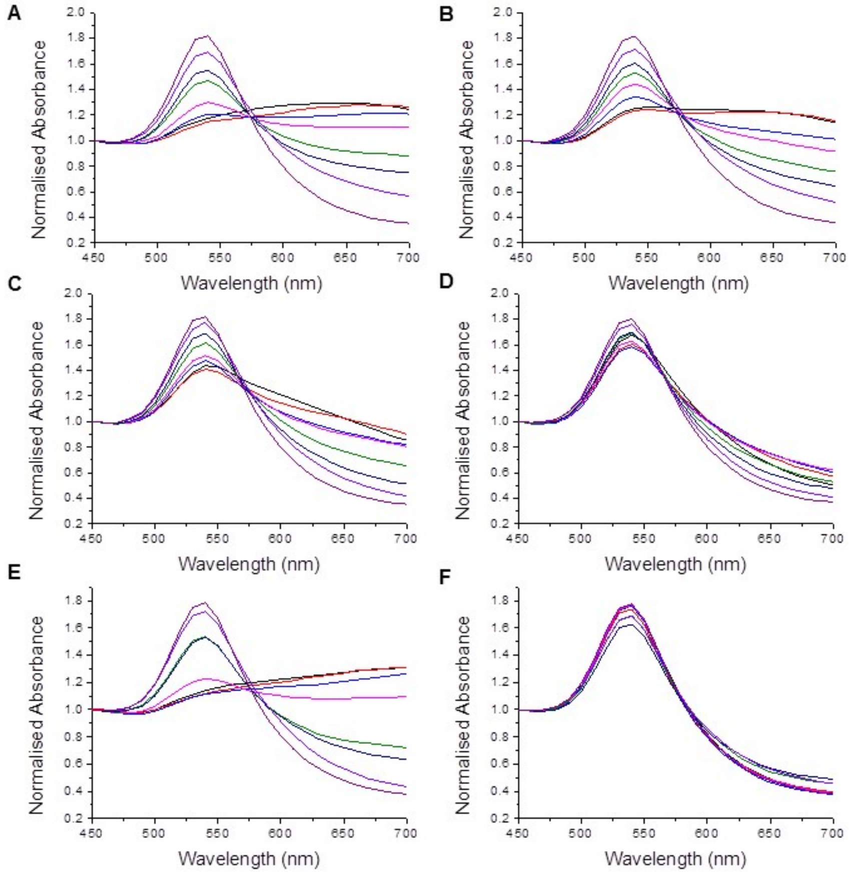

Figure S7: Glc-AuNPs with A) Con A, B) RCA, C) SBA, D) PNA, E) WGA, F) UEA.

N-acetylmannosamine functional particles

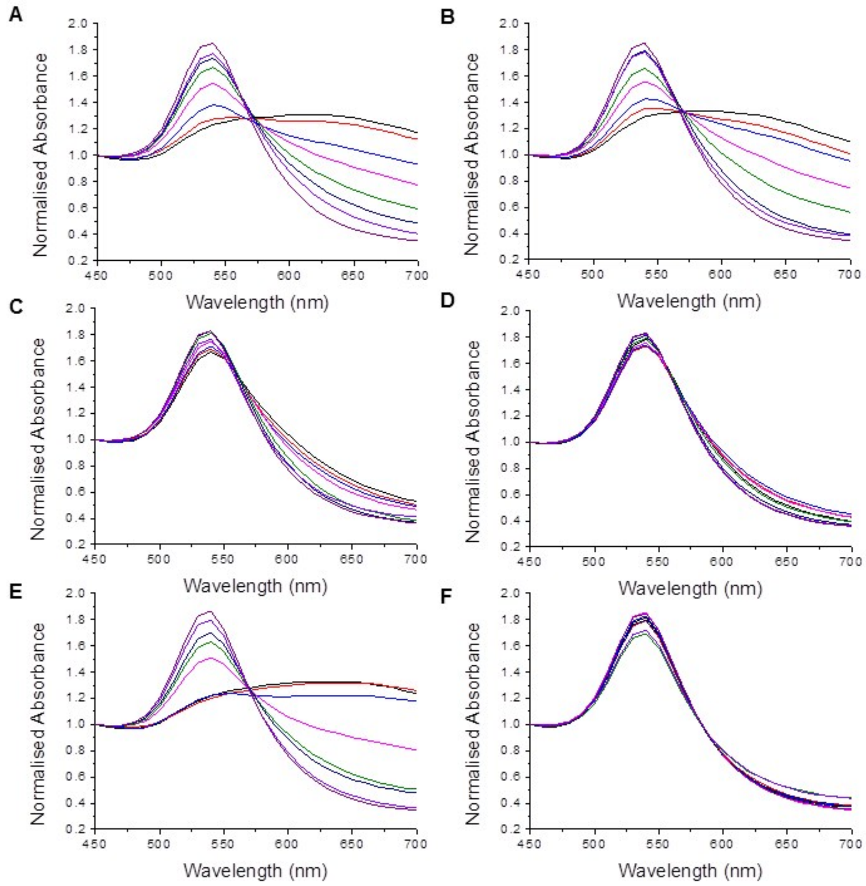

Figure S8: ManNAc-AuNPs with A) Con A, B) RCA, C) SBA, D) PNA, E) WGA, F) UEA.

N-acetylgalactosamine functional particles

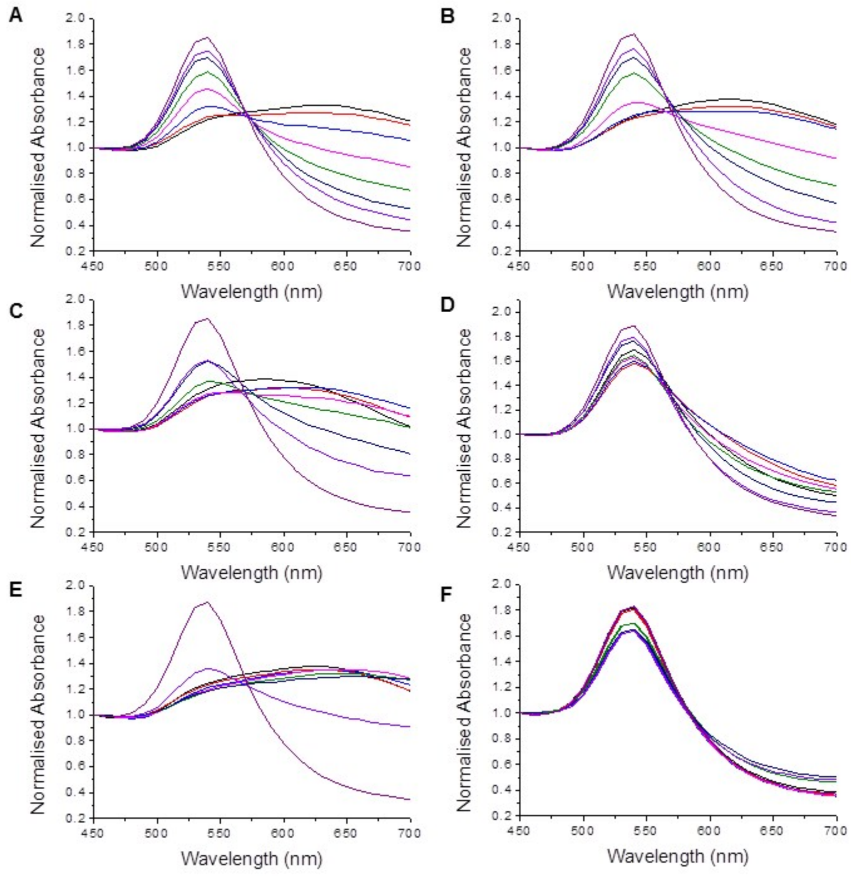

Figure S9: GalNAc-AuNPs with A) Con A, B) RCA, C) SBA, D) PNA, E) WGA, F) UEA.

N-acetylglucosamine functional particles

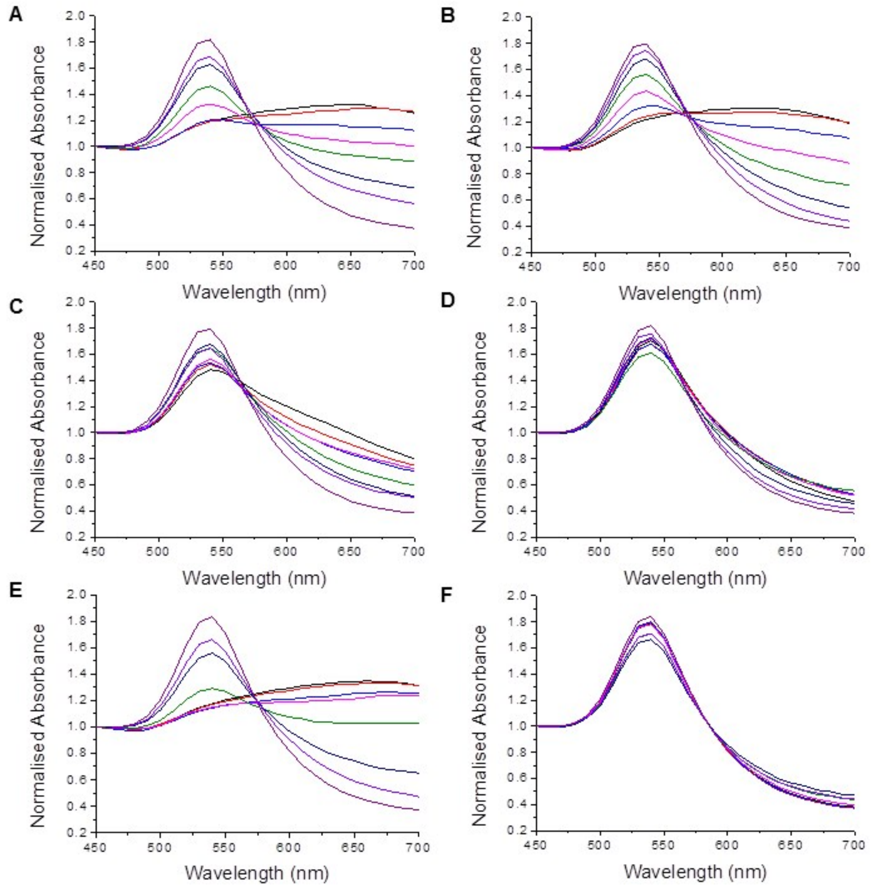

Figure S10: GlcNAc-AuNPs with A) Con A, B) RCA, C) SBA, D) PNA, E) WGA, F) UEA.

Fucose functional particles

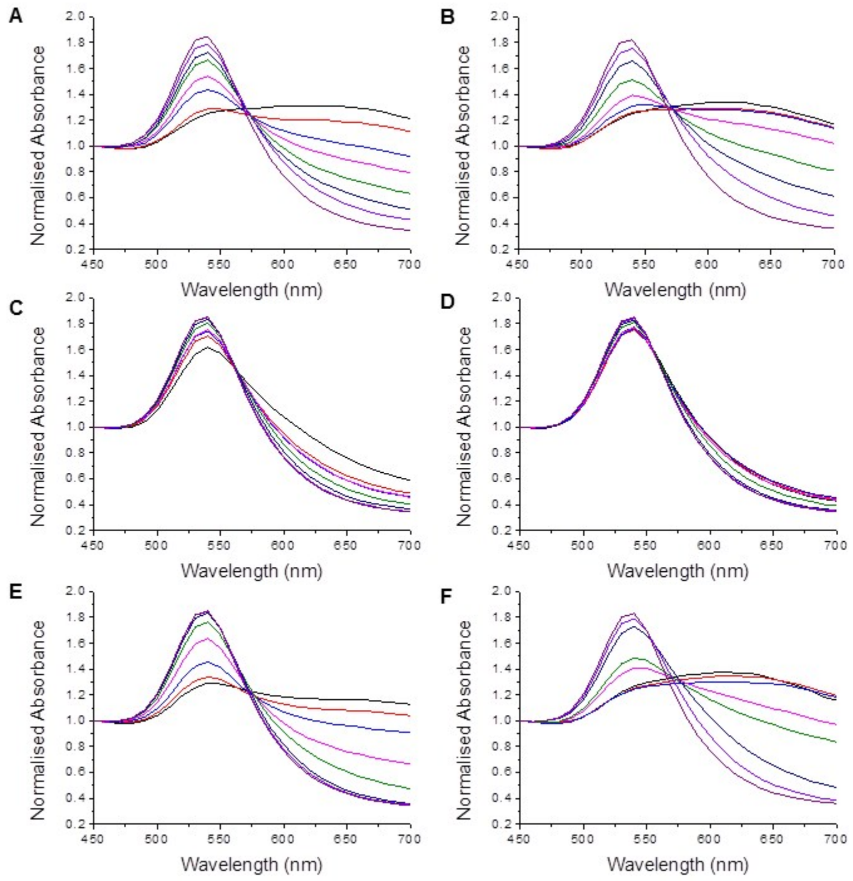

Figure S11: Fuc-AuNPs with A) Con A, B) RCA, C) SBA, D) PNA, E) WGA, F) UEA.

Determining apparent K_d values using a image J to determine pixel intensity of a scanned image of the 96 well plate.

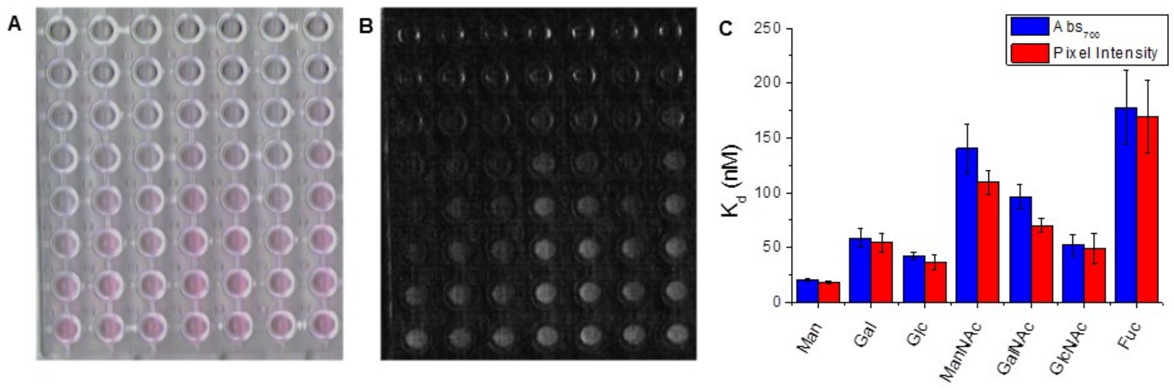

Figure S12: Direct optical analysis of Con A binding. A) Scanned image of glycoAuNPs with a dilution series of Con A after 30 minutes. (B) Saturation image used for pixel intensity measurement (high intensity = red, low intensity = blue). C) Comparison of K_d calculated by Abs_{700} and pixel intensity.

Correlation between Kd determined by absorbance and by using pixel intensity determined from a scanned image of the 96-well plate for Con A, RCA, SBA and WGA.

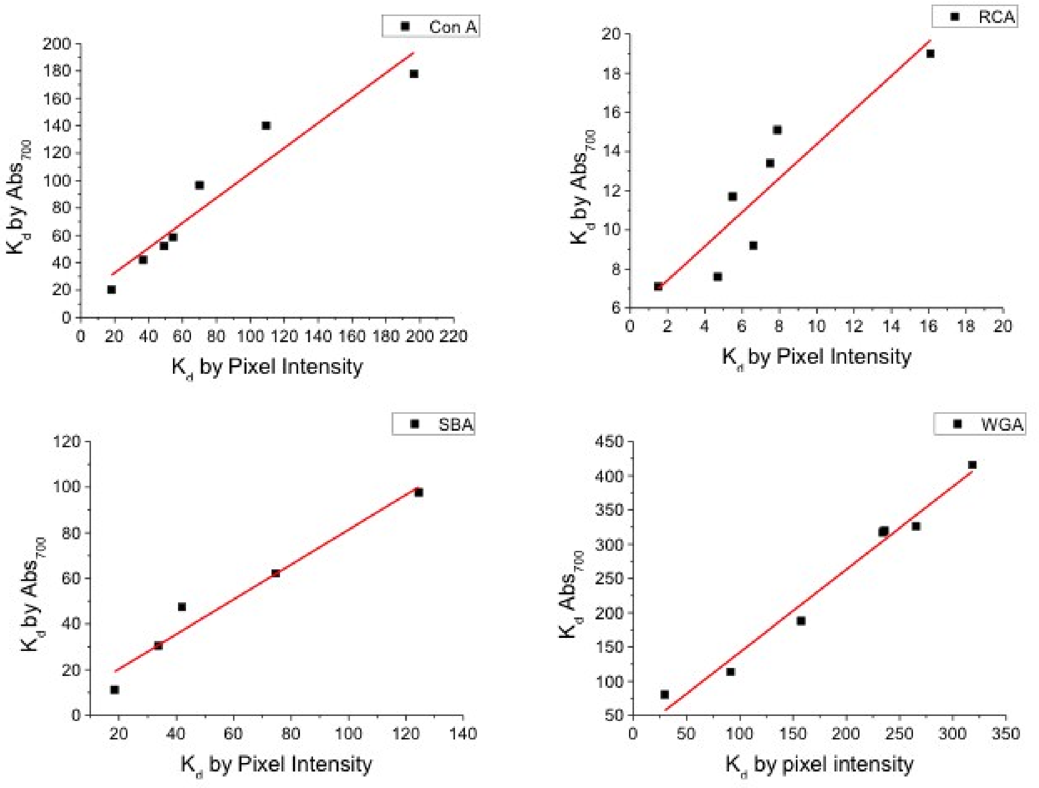

Figure S13: Correlation between K_d values obtained from Abs_{700} measurements using a uv/vis plate reader vs. pixel intensity from a scanned image.
